# From the Consulting Room to the Court Room? Taking the Clinical Model of Responsibility Without Blame into the Legal Realm

**DOI:** 10.1093/ojls/gqs028

**Published:** 2012-11-19

**Authors:** Nicola Lacey, Hanna Pickard

**Keywords:** blame, desert, justice, agency, punishment, rehabilitation, responsibility, treatment

## Abstract

Within contemporary penal philosophy, the view that punishment can only be justified if the offender is a *moral agent* who is responsible and hence blameworthy for their offence is one of the few areas on which a consensus prevails. In recent literature, this precept is associated with the retributive tradition, in the modern form of ‘just deserts’. Turning its back on the rehabilitative ideal, this tradition forges a strong association between the justification of punishment, the attribution of responsible agency in relation to the offence, and the appropriateness of blame. By contrast, effective clinical treatment of disorders of agency employs a conceptual framework in which ideas of responsibility and blameworthiness are clearly separated from what we call ‘affective blame’: the range of hostile, negative attitudes and emotions that are typical human responses to criminal or immoral conduct. We argue that taking this clinical model of ‘responsibility without blame’ into the legal realm offers new possibilities. Theoretically, it allows for the reconciliation of the idea of ‘just deserts’ with a rehabilitative ideal in penal philosophy. Punishment can be reconceived as consequences—typically negative but occasionally not, so long as they are serious and appropriate to the crime and the context—imposed in response to, by reason of, and in proportion to responsibility and blameworthiness, but without the hard treatment and stigma typical of affective blame. Practically, it suggests how sentencing and punishment can better avoid affective blame and instead further rehabilitative and related ends, while yet serving the demands of justice.

The degree of civilization in a society can be judged by entering its prisons.

—Fyodor Dostoyevsky

## 1. Introduction

Within contemporary penal philosophy, the view that punishment can only be justified if the offender is a *moral agent* who is responsible and hence blameworthy for their offence is one of the few areas on which a consensus prevails. In recent literature, this precept is associated with the retributive tradition, in the modern form usually known as the ‘just deserts’ or ‘justice’ model. On this model, punishment is hard treatment which is visited on the offender in response to, by reason of, and in proportion to his or her blameworthy conduct.^1^ Blameworthy conduct, in turn, demands that the offender have the capacity for responsible agency: minimally, cognitive and volitional capacities such that they know what they are doing when they commit an offence, and exercise choice and a sufficient degree of control in doing so. Turning its back decisively on the rehabilitative ideal characteristic of penal philosophy in the 1960s, this tradition forges a strong association between the justification of punishment, the attribution of responsible agency in relation to the offence, and the appropriateness of blame.

In this article, we offer an alternative model that challenges the strong association between punishment and blame, while nonetheless retaining the emphasis on the offender’s capacity for responsible agency. The model draws on the nature of effective clinical treatment of patients with disorders of agency that involve wrongdoing or cause harm, such as certain personality disorders, impulse-control disorders and addictions. Core diagnostic symptoms of such disorders include actions and omissions that are criminal or morally wrong.^2^ But evidence-based treatment for these conditions typically depends on clinicians adopting a stance towards patients of ‘responsibility without blame’.^3^ This clinical stance implicitly employs a conceptual framework in which ideas of responsibility and blameworthiness are clearly separated from what we shall call ‘affective blame’, together with the capacity to implement this framework in practice.^4^ Affective blame, as we define it, is the range of hostile, negative attitudes and emotions that are typical human responses to blameworthiness. It can include, for instance, hatred, anger, resentment, indignation, disgust, disapproval, contempt and scorn, and can be manifest in any number of ways, including seeking retaliation, retribution, and vengeance, rejection and banishment from the community, and the withdrawal of basic respect. In the face of culpable wrongdoing or harm, we often feel such hostile, negative attitudes and emotions are justified and appropriate: that we are *entitled* to feel and act in these ways, because of what the person in question has done—they deserve it.^5^ In keeping with the justice model, the clinical model judges patients responsible and indeed accountable for wrongful or harmful conduct to the extent that they possess the relevant cognitive and volitional capacities in relation to it. But in contrast, it resists any corresponding tendency towards affective blame. Put simply, according to the clinical model, blameworthiness, understood as responsibility and accountability for wrongdoing, does not entail the ‘worthiness’ of affective blame.

We argue that taking this clinical model of responsibility without affective blame into the legal realm suggests new theoretical and practical possibilities. Theoretically, it becomes possible to reconceive punishment as at one and the same time justified by responsibility for blameworthy conduct, but severed from affective blame. Punishment can be understood simply as the imposition of consequences—typically negative but occasionally not, so long as they are serious and appropriate to the crime and the context—in response to blameworthiness for wrongdoing or harm. Punishment need not be imposed *out of or in connection with affective blame.* This severance from blame may facilitate the use of punishment for therapeutic ends, thereby allowing the theoretical integration of the justice model with the rehabilitative ideal: although punishment may be justified by blameworthy conduct, its purposes may—indeed, where possible, should—include rehabilitation. Practically, although this re-conception of punishment leaves the grounds for conviction unchanged, it allows us to reconsider sentencing procedures in the courts, and the nature and execution of punishment—and its aftermath—in prison and in the community. For, according to our alternative model, rehabilitation need not entail the effacement of moral responsibility, and justice need not entail the hard treatment and stigma that is typical of affective blame, even when negative consequences are justified and imposed.

The article proceeds as follows. In the first section, we briefly sketch the post-war history of both philosophical and policy debates about moral agency and blameworthiness in the criminal justice context. We focus in particular on the influence of the rehabilitative ideal during the post-war era, and on the reasons for the reaction against this ideal and in favour of a revival of retributive theory in the modernized form of ‘just deserts’. We note that this reaction was underpinned not merely by doubts about the efficacy of rehabilitative programmes, but by concern that the rehabilitative ideal failed to acknowledge the moral agency of offenders and so eroded their responsibility because of its conceptualization of crime as symptomatic of pathology in need of treatment. And we show that the retributive revival within academic penal philosophy has too often proceeded on the assumption that the centrality of responsible agency to the justification of punishment implies the appropriateness of the hard treatment and stigma that is part and parcel of affective blame.

In the second section of the article, we describe the clinical stance of responsibility without blame in more detail, and argue that even when crime or immorality is symptomatic of pathology and can be effectively treated, this need not imply the effacement of moral agency. On the contrary, we argue that the best understanding of the therapeutic process sets responsibility at the core of clinical practice: treatment demands that clinicians and patients alike presume that patients can decide to change maladaptive patterns of behaviour, including those that are criminal or immoral, and so have choice and a sufficient degree of control to do so. Indeed, for treatment to be effective, clinicians must hold patients responsible and indeed accountable for their blameworthy conduct as a way of engaging and developing moral agency.^6^ The key is to do so while avoiding the range of hostile, negative attitudes and emotions that constitute affective blame and which we can feel that people who act criminally or immorally deserve, and instead maintaining throughout human concern, respect and compassion.

In the third section of the article, we consider some of the aims and procedures employed in clinical contexts that help to keep affective blame at bay. We explore both the similarities and the differences between the clinic and the courtroom, and, correspondingly, the extent to which it is feasible for the courts to adopt comparable aims and procedures to the clinic, particularly at the sentencing stage of the justice process. In so doing, we argue that we not only have good instrumental reasons to take the clinical model into this aspect of the legal realm, but may indeed, as a society, have a moral obligation to do so.

In the fourth part of the paper, we consider the effect that affective blame can have within institutions that punish, such as prisons, and on the attitudes towards and demands placed on offenders when they have served their sentence and return to the community. In the fifth and final section, we conclude with a brief summary of the argument presented.

The appeal of the justice model lies fundamentally in the force of the intuition that punishment is only justified if the offender has the capacity for responsible agency and hence is blameworthy for wrongdoing or harm perpetrated. Although this intuition is both deep and pervasive in our common morality, there are, of course, other models for the justification of punishment. Although we recognize the force of the intuition behind the justice model, we do not here commit ourselves to its truth.^7^ Rather, our aim is to show that, even *if* the force of the intuition is granted and the justice model adopted, it does not entail that punishment demands hard treatment and stigma, in the form of affective blame and corresponding penal practices. This opens the door to the affirmation of the basic moral and political values inherent in a broadly liberal, democratic society, where respect and equality ideally accrue to all. Even if we grant that punishment is justified only when responsive to the offender’s moral agency, these values demand that it should nonetheless proceed not only with humanity and dignity, but with a view towards rehabilitation and reintegration of offenders into the moral community.

## 2. The History of British and American Penal Theory and Practice in the Post-War Era

Although the intuition that punishment is only justified if the offender has the capacity for responsible agency which underpins the justice model has great force, the consensus surrounding it within penal theory and practice has emerged within a distinctive historical context. After World War II, particularly in countries like Britain and the United States, a variety of consequentialism, widely known as the rehabilitative ideal, gained ground as a rationale for state punishment. According to the rehabilitative ideal, punishment was justifiable simply by appeal to its potentially rehabilitative consequences. Reform of the offender had, of course, long sat alongside other consequentialist goals, such as deterrence and incapacitation. But the particular form of the post-war rehabilitative ideal threw into sharp relief a difficulty about how punishment could in principle be rendered compatible with adequate respect for the individual as a responsible moral agent.

Quite generally, purely consequentialist theories appear to lack the resources to explain our intuition that certain acts are impermissible—a gross injustice and violation of the duty to respect the rights of the individual—no matter what their consequences. For instance, it is wrong to maximize good for society as a whole by excessive punishment of individual offenders, never mind by such extremes as punishment of the innocent, pre-emptive ‘treatment’ of the ‘dangerous’, or criminalization of those not fully responsible for their actions. To put this objection in the Kantian terms to which John Rawls’ work gave such a marked revival in the early 1970s,^8^ such practices amount to treating individuals merely as means rather than as ends in themselves.

The rehabilitative ideal in punishment encountered this difficulty in particularly stark terms. For, in its purest, theoretical form^9^ it entailed, as HLA Hart put it, an ‘elimination of responsibility’^10^—indeed, perhaps of anything recognizable as ‘punishment’—in favour of a model not of blameworthiness and sanction but rather of diagnosis and treatment.^11^ Criminal conduct was to be understood as a symptom of some underlying individual or social pathology. Of course, *causal* responsibility for such conduct still needed to be established at trial: the right person must be convicted. But there was, according to this pure, theoretical form of the rehabilitative ideal, no need to establish that the offender had the requisite cognitive and volitional capacities for responsible agency: that they knew what they were doing at the time of the offence, and exercised choice and a sufficient degree of control in doing so. Indeed, responsible agency of this more demanding kind was beside the point, which was quite simply to find means of rehabilitating offenders, and thereby reducing crime, through clinical interventions of both medical and behavioural kinds.

In practice, the rehabilitative ideal did not affect the conditions necessary for a court’s finding of criminal liability: it has always been necessary for the offender to be shown to have been a responsible agent at the time of the offence. However, to varying degrees and in various ways, it did affect the practice of sentencing and the execution of punishment. At the sentencing stage, criminal acts and omissions tended to be regarded as symptoms of an underlying pathology which was assumed to contribute causally to the criminal misconduct. It was therefore natural to understand such behaviour as by-passing the individual’s cognitive and volitional capacities, belying the finding of criminality liability, and undermining the presumption of genuine individual choice or control: this is what it means for criminal misconduct to be understood as a manifestation of individual or social disease. There was, then, at the sentencing stage of the criminal process as well as during its aftermath, little scope for responding to offenders as responsible and moral agents and thereby according them the rights and respect—alongside punishment for blameworthy conduct—appropriate to that agency. Rather, they all too easily became a problem to be managed and controlled.

In practice, this had two consequences. On the one hand, it led to a rise in indeterminate sentences based on predictions of ‘dangerousness’ or need for treatment. On the other, it allowed broad and unaccountable official discretion as to release date, based on expert judgements about prognosis, risk and ‘cure’. It is little wonder, then, that given the excesses and injustices associated with this model, philosophers and policy-makers alike recoiled from it, turning instead towards a revival of retributive theory under the new label of ‘just deserts’.^12^ The concern to establish respect for responsibility and moral agency as core values of the criminal justice process was not, of course, restricted to proponents of ‘just deserts’:^13^ it also characterized theories which seek to combine backward-looking and forward-looking considerations in the justification of punishment.^14^ But the justice model both captured the imagination of policy-makers in a number of western countries, and represented itself as the only approach capable of generating an account of punishment compatible with full respect for offenders as responsible and moral agents. It is accordingly on this argument that we focus our attention.

It is undoubtedly the case that the reaction against the rehabilitative ideal was based on an exaggerated view of how widely spread or fully realized its more radical manifestations were.^15^ But the important point for our purposes is that its demise was premised not only on the failure of rehabilitative programmes to reduce crime,^16^ but also on revulsion at the failure to respect the offender’s moral agency. Moreover, and crucially, it was this concern about respect for moral agency that allowed proponents of what, in the immediate post-war era, had been widely regarded as a severe, perhaps even atavistic, view of punishment as retribution, to reinvent itself as the view most closely aligned with moderation and a respect for civil rights.^17^ On the justice model, punishment is justified in response to, by reason of, and in proportion to, the offender’s desert. Desert in turn is premised on his or her blameworthiness, which is generally understood in terms of a combination of wrongful or harmful conduct and culpability for that conduct. Central to culpability, as we have emphasized, is the fact that the offender has normal volitional and cognitive capacities which were adequately engaged at the time of the offence: they knew what they were doing when they committed the crime, and exercised choice and a sufficient degree of control in doing so. By linking not only the justification but the distribution and quantum of punishment to the offender’s desert, in the sense of the level of blameworthiness appropriately to be attached to the offence, the justice model purported to offer a clear *limit* on state punishment. It thus presented itself as a progressive, humane, and even liberal approach, with respect for the offender’s personhood, as manifest in their capacity for responsible agency and morality, at the core of its moral vision.

Thirty years on, however, the practical impact of the justice model presents a very mixed picture in terms of both effective limits on punishment and real respect for offenders as persons. Several of the countries in which it has had the most decisive influence on policy—notably Britain and the United States—have in fact seen an upswing in overall severity of sentences during this era, and a continuation or even acceleration of practices, such as indeterminate sentencing and preventive justice, which were thought to express the more extreme disrespect for the rights and agency of the offender that characterized the rehabilitative ideal, in principle and in practice.^18^ Of course, it would be wrong to make any strong claims about causation here: many factors are involved in shaping levels of punishment. They include trends in both crime and fear of crime, the institutional structures of the political systems which shape penal policy, and a variety of other social, political and economic factors.^19^ The precise shape of the sentencing system through which different societies and regions have attempted to implement the justice model is another important variable.^20^ Indeed, not all the countries that institutionalized the justice model in terms of sentencing reforms have seen a significant increase in punishment, with Sweden a key example in this respect.^21^ Moreover, proponents of the justice model vary in the degree to which they espouse a commitment to blame and desert as distinct from proportionality, inviting the argument that politicians have not so much adopted as distorted the model, or have adopted it in extreme forms which most of its advocates reject.^22^ Nonetheless, recent influential statements of the justice model within academic penal philosophy have come increasingly to emphasize the hard treatment and stigmatization typical of affective blame as components of a ‘just deserts’ conception of punishment.

For example, notwithstanding his explicit commitment to promoting reform and reconciliation, Duff insists that hard treatment is intrinsic to the communicative theory of punishment.^23^ As Matt Matravers^24^ has convincingly shown, this aspect of his theory is not fully justified by the contours of Duff’s argument. The fact that it holds a central place while being incompletely justified risks that an approach which explicitly aspires to foster inclusiveness may be read as inviting exclusionary punitiveness.^25^ Similarly, Duff’s view of mercy as extrinsic, rather than, as John Tasioulas^26^ has argued, intrinsic to penal justice, marks even this most humane and liberal version of the justice model as liable to foster exclusionary punitive dispositions. And while von Hirsch’s theory does not assume that punishment must involve hard treatment, the idea of ‘censure’ to which he appeals both attaches more naturally to persons than to conduct, and evokes the flavour of affective blame while lacking any explicit renunciation of its appropriateness. Without due caution against its potential excesses, this notion of ‘censure’ may not entail, yet invites, the possibility of lasting judgement and stigmatization of persons.^27^ Other recent contributions to penal philosophy in the broadly retributive tradition have gone further, explicitly embracing the idea that punishment is intrinsically exclusionary or stigmatizing. For example, in a recent paper Douglas Husak argues that ‘a state response to conduct does not qualify as punitive unless it is designed to censure *and to stigmatize*’ (our emphasis).^28^ And Daniel McDermott^29^ argues explicitly for the essentially exclusionary dynamic of retribution, and regards imprisonment as a presumptively acceptable penalty on the basis that, in the style of banishment, it excludes wrongdoers from the moral community. Note that this is an argument that sidelines the potentially rehabilitative and reintegrative aspects of imprisonment, hence placing it on a spectrum not only with banishment, but also with forms of penalty such as branding and capital punishment.

There are, of course, important differences between these versions of the justice model, which appeal to various concepts to articulate the idea of punishment, including not only hardness of treatment and stigma, but also censure, exclusion and absence of mercy. It is an open question whether and to what extent these different concepts can be divorced from affective blame and so married to the reconception of punishment that we propose here. Our point is that, in the absence of a clear account of the nature of affective blame and an explicit rejection of its appropriateness, all these versions of the justice model, notwithstanding their avowed respect for agency and for limits on punishment, naturally evoke affective blame and in doing so lend moral imprimatur to hard treatment and stigma as intrinsic to legitimate punishment. In so far as intellectual trends can have practical consequences, this may have contributed to the current vehemence of many popular demands for severity in punishment and the willingness of criminal justice officials to meet them. In a world where measures of retribution grounded in symbols which command wide consensus no longer guide the match between punishment and crime—a world, in short, far distant from that of the *lex talionis*—there is no simple mechanism for anchoring the scale and nature of the penalty to ‘cardinal proportionality’. In this vacuum, particularly under conditions of a highly politicized climate for criminal justice policy-making,^30^ the commitment to ‘just deserts’ can produce insatiable demands for vengeance: ‘hard treatment’ all too easily becomes ‘bad treatment’. In public discourse about crime, there is an insistent worry that our capacity to hold offenders responsible and accountable for misconduct is threatened by an attitude of concern, respect and compassion. For it may seem as if these attitudes can tempt us to excuse offenders from responsibility: to hold offenders responsible, we must respond with treatment which is hard and stigmatizes—or censures, excludes or shows no mercy.

In the next section, we show that although this line of thought may be natural, it is nonetheless possible to strike a balance: reflection on effective clinical treatment of disorders of agency demonstrates that concern, respect and compassion need not block the attribution of responsibility and the demand for accountability. Disorders of agency do not render otiose individual choice and control: the conditions for responsible agency are to a sufficient degree intact despite the presence of pathology, and indeed must be targeted if treatment is to be effective. Nonetheless, if treatment is to be effective, it must proceed without affective blame. The clinical context thus offers a model for how punishment, understood as the imposition of consequences—typically negative but occasionally not, so long as they are serious and appropriate to the behaviour—can be imposed in response to blameworthy conduct, without the offender being thereby subject to hard treatment and stigma, or censured, excluded from the community, or shown no mercy. Rather, punishment can proceed not out of or in connection with affective blame, but hand in hand with concern, respect and compassion. In essence, the clinical model offers a corrective to the potential punitive excesses which have emerged over the past decades, both in theory and in practice, as part and parcel of the justice model.

## 3. A Conceptual Framework for Responsibility Without Blame

The clinical stance of responsibility without blame is central to effective treatment of disorders of agency. Disorders of agency are psychiatric disorders, such as personality disorders, impulse-control disorders and addictions, where core diagnostic symptoms include actions and omissions: patterns of voluntary behaviour central to the nature or maintenance of the condition. For instance, borderline personality disorder is diagnosed in part via deliberate self-harm and attempted suicide, reckless and impulsive behaviour, substance abuse, violence and outbursts of anger; antisocial personality disorder is diagnosed in part via criminal behaviour, alongside disrespect for, and violation of, the rights of others; addictions are diagnosed via maladaptive patterns of alcohol and drug consumption or other problematic behaviours, which may be criminal, and lead to severe negative consequences for self and others. As these examples make plain, many of the actions and omissions that are central to disorders of agency count as criminal or immoral: they often involve wrongdoing and typically cause significant harm, for self and others. It is perhaps no surprise, then, given the moral and indeed criminal component of the pathological behaviour, that 64% of male and 50% of female offenders have a personality disorder.^31^ Indeed, in many such cases, one and the same kind of behaviour is both treated by clinicians, and prosecuted by the criminal courts. When this is so, psychiatric improvement, rehabilitation and reform must necessarily proceed hand in hand. For, given the nature of disorders of agency, psychiatric improvement, let alone recovery, requires there to be a change in the diagnostic pattern of behaviour. When this pattern involves criminal or immoral behaviour, psychiatric improvement *just is* moral improvement and reduced criminality.

Although medication can be helpful for treatment of such disorders,^32^ the voluntary nature of the behaviour means that the power to change it nonetheless lies fundamentally with the patient. For instance, if they are to improve or recover, patients with borderline personality disorder must stop self-harming; patients with antisocial personality disorder must stop breaking the law; addicts need to quit using drugs or alcohol. There are, no doubt, equally central cognitive and affective components to disorders of agency. Borderline personality disorder involves instability of self-image and emotional volatility; antisocial personality disorder involves lack of remorse; addicts may use drugs and alcohol, for instance, to deal with psychological distress, and so abstinence may be easier if that distress is addressed and otherwise managed.^33^ Nonetheless, because actions and omissions are diagnostically central to disorders of agency, effective clinical treatment must address these voluntary patterns of behaviour, even if outcomes are improved by an integrative approach that treats behaviour alongside cognition and affect.

A central component of treatment is therefore to engage and develop the patient’s capacity for responsible agency. All currently favoured psychological treatments^34^ are united in presuming that patients have the capacity for choice and control over maladaptive behaviour to a significant degree, and that the clinical aim is, at least in part, to motivate, encourage and support the patient to do things differently by choosing to alter entrenched maladaptive patterns, and effecting this choice even in the face of inclinations to revert to old habits.^35^ These therapies differ simply in the extent to which this presumption of responsible agency is explicitly part of how the clinician engages the patient. For instance, in motivational interviewing, the clinician adopts a non-challenging stance, expressing empathy and encouraging the patient to see the unwanted consequences of their behaviour. In contrast, the language of agency and responsibility permeates the culture of Therapeutic Communities—including those run for offenders within prisons such as Grendon.^36^ It is an expectation of Therapeutic Communities that their members see themselves and others as responsible agents: accountable for their behaviour and capable of change. But, in all these cases, it is a presumption of effective treatment that patients have choice and a significant degree of control over their behaviour and can therefore be asked to take responsibility for it, as we naturally say: their cognitive and volitional capacities are, even if on occasion reduced compared to the norm, intact to a sufficient degree for agency and responsibility to be appropriately attributed and engaged.^37^

Part of asking patients to take responsibility for behaviour involves pointing out or indeed imposing consequences should they fail. Most minimally, motivational interviewing proceeds by emphasizing the unwanted consequences to the patient of their behaviour, in the hope that these will act as a natural deterrent, motivating the patient to change. Within other forms of therapy, such as emotional intelligence and mentalization-based therapy, clinicians or, if group-based, other patients, may offer challenging feedback, so that the negative effects of problematic behaviour on self, others and relationships is made explicit to the patient, and cannot be avoided or denied by them. Finally, and most robustly, varieties of cognitive-behavioural therapy and Therapeutic Communities employ rules and contracts between patient and clinician or patient and group, whereby negative consequences are imposed by the clinician or group (typically with advance warning and the agreement of the patient) if patients fail to change problematic behaviour. Although these consequences typically involve a reflective component to encourage patients’ understanding of why they lapsed on this occasion, and to develop a plan for how to succeed next time, they may also involve measures that are more potentially punitive, such as withdrawal of privileges, or time-limited suspension from the group. Most forms of effective therapy thus involve not only responsibility but accountability: a demand that patients must answer and explain themselves to clinician or group, together with the imposition of negative consequences, if they fail to change problematic behaviour.

It is a staple of clinical practice that, because these negative consequences are potentially punitive, they must be effected with an attitude of concern, respect and compassion, as opposed to blame. But, as with much clinical practice, questions remain as to what exactly this means, and why it should be true. On the one hand, a patient’s experience of how they are treated may diverge from a clinician's or group’s experience of how they are treating a patient, inviting the question of who gets to decide what in fact happened—for instance, whether or not a patient has been stigmatized or blamed. However, in practice, the procedure for handling such cases of disagreement is clear. The attitude of concern, respect and compassion that pervades treatment dictates that, at a minimum, the clinician or group acknowledges the patient’s experience, reflects on their own attitudes and behaviour and how they may impact on the particular psychology of the patient, and aims to work towards a shared understanding of the therapeutic process and what in fact occurred, as a basis upon which to proceed. In essence, a commitment to dialogue, reflection and negotiation in the face of disagreement is part of what it is to treat patients with concern, compassion, and respect within the clinic—even if the disagreement cannot in the end be overcome and a shared understanding of the meaning of an interaction achieved.

On the other hand, there is as yet no fully established answer to the question of why it should be that, although responsibility and accountability are central to effective treatment, blame, in contrast, is detrimental. There is research that suggests a compassionate attitude has a positive therapeutic effect^38^ but no independent research, to our knowledge, that suggests that a blaming attitude has a negative effect. Additionally, the mechanisms by which these attitudes, compassionate or blaming, effect therapeutic outcomes are as yet untested. It is natural to speculate that blaming patients may trigger feelings of rejection, anger, and self-blame, which bring heightened risk of disengagement from treatment, distrust and breach of the therapeutic alliance, relapse, and, especially with patients with personality disorders, potentially even self-harm or attempts at suicide. But such speculation, however natural, represents a very general empirical hypothesis that only experimental psychology can refine and assess.^39^ Until that research is undertaken, the clinical staple that blame must be avoided remains an integral part of clinical practice, but without a full explanation of *why* this should be.

However, for the purposes of this article, we can proceed in absence of such an explanation. Nobody likes to be blamed. We all have some grip on what it is like to feel the object of another’s hostile, negative attitudes and emotions, and why this might be detrimental to one’s sense of self-worth, one’s relationships with others, and one’s hopes for the future and motivation to change aspects of oneself that are difficult to face. What the clinical model offers is a clear and well-established practice of holding people responsible and indeed accountable for criminal or immoral conduct without affective blame, but with concern, respect, and compassion. The rest of this section articulates the conceptual framework implicit in this practice, distinguishing clearly between our concepts of responsibility, blameworthiness and affective blame.

### A. Responsibility

Within philosophy, as well arguably as the law and society at large, there is a deep-rooted tendency to link our idea of responsibility fundamentally with morality, by holding that its point or purpose is *moral evaluation*: the assessment of another and their behaviour as good or bad, right or wrong. This link can be more or less strong. At its strongest, philosophers such as PF Strawson^40^ and followers argue that our concept of responsibility is grounded in our practice of holding others responsible via our ‘reactive attitudes’ or ‘moral emotions’, such as forgiveness, gratitude, indignation, resentment, and, most importantly for our purposes, blame. Though it is unclear how exactly Strawson himself conceives of the link between responsibility and such attitudes, it is often taken to be constitutive. As Gary Watson expresses this view: ‘to regard oneself or another as responsible just is the proneness to react to them in these kinds of ways’.^41^ Others have argued that to hold another responsible is to believe that reactive attitudes are *appropriate, fitting* or *deserved* responses to their behaviour, even if one does not actually feel anything oneself.^42^ Either way, the concept of responsibility is essentially linked to our moral attitudes and emotions towards others, including blame. In contrast, philosophers sometimes link responsibility with morality more weakly, simply by using the concepts of responsibility and moral responsibility interchangeably. For instance, Derk Pereboom opposes Strawson and followers by arguing that there must be room for a person to be ‘morally responsible for an action even if she does not deserve blame, credit, or praise for it—if, for example, the action is morally indifferent’.^43^ He thus severs the link between responsibility and our reactive attitudes. But the link between responsibility and morality is yet, by implication, preserved. For the kind of responsibility attributed for even morally indifferent actions is still *moral* responsibility. Morality remains the ultimate point or purpose of the idea.^44^

This tendency to link responsibility with overall moral evaluation is incompatible with the demands of effective clinical treatment. On the one hand, in its stronger form, it renders the clinical stance of holding patients responsible for criminal or immoral behaviour without blaming them close to conceptually incoherent. If holding someone responsible for harm just is responding with a reactive attitude like blame, then it is not possible to hold patients responsible for harm without blaming them. Similarly, if holding someone responsible for harm just is believing that blame would be an appropriate, fitting or deserved response, then, although one may not oneself be blaming them, one is hardly adopting the blame-free stance which is necessary for effective clinical treatment. In practice, one might as well be blaming them, for without further qualification, one believes that one should. In essence, a view of responsibility that links it so closely to the reactive attitudes cannot meet the demand inherent in clinical treatment that it be possible to hold patients responsible for wrongful or harmful behaviour, without blaming them for it. For according to such a view, blaming is too much a part of what it means to hold another responsible for there to be sufficient room to manoeuvre between them.

On the other hand, in its weaker form, this tendency to link responsibility with moral evaluation risks the implication that patients are subject to *moral evaluation* by clinicians when they are explicitly encouraged to take responsibility for problematic behaviour, inviting a subjective experience of being the object of blame. This risk is no doubt heightened when the behaviour in question is indeed criminal or immoral. But it exists even in contexts where the behaviour targeted by clinical treatment is morally indifferent and problematic only from the point of view of the patient’s own mental health and wellbeing: patients are often prone to feel deep shame and guilt about many aspects of themselves, and to hold core beliefs that they are ‘bad’ or ‘wrong’ which are easily triggered.^45^

In the clinic, the point or purpose of the idea of responsibility is not, contra philosophical accounts, moral evaluation. The point or purpose is motivation and capacity to change. For, once again, patients with disorders of agency must change patterns of actions and omissions if they are to improve or recover. Responsibility is therefore fundamentally linked, not to the reactive attitudes or morality, but quite simply to agency: to having power over one’s behaviour. Importantly, this idea of responsibility is neither rare nor novel. Rather, it is rooted in common sense, the history of philosophy, and, as we saw above in discussing the conditions for responsible agency, the law itself.^46^

Our common sense conception of agency draws a basic distinction between actions and mere bodily movements, such as automatic reflexes. What makes a piece of behaviour an action, as opposed to a mere bodily movement, is that it is voluntary, where this means that the agent can exercise choice and at least a degree of control over the behaviour. Within philosophy, this conception of agency is traditionally linked to the idea of free will, and can arguably be found in philosophers as diverse as Aristotle,^47^ Hobbes,^48^ Hume,^49^ Reid^50^ and Kant.^51^^,^^52^ Within the law, this conception of agency is necessary for criminal liability. According to this shared conception, agency requires two capacities: first, the capacity to choose from a range of possible actions, at least in the minimal sense that, on any particular occasion, one can choose either to act, or to refrain from so acting; second, the capacity to execute this choice: to do as one chooses, given normal circumstances.^53^ This common sense conception of agency naturally grounds judgements of responsibility: one is responsible for actions, as opposed to automatic reflexes, because it is up to one whether and how one acts. So long as one knows what one is doing, one is responsible for one’s behaviour to the degree that one can exercise choice and control over it. Crucially, we are therefore responsible for our actions (or omissions) in so far as we are their agents: our behaviour is a manifestation of the requisite cognitive and volitional capacities.

Of course, the link between responsibility and agency is not quite this simple. Both in the clinic and in the courts, there can be justifications and excuses for wrongful or harmful conduct which affect responsibility and blameworthiness,^54^ and hence also the nature of appropriate treatment and judgements of criminal liability. Equally, just as the cognitive and volitional capacities required for attributions of responsibility are not all-or-nothing but come in degrees, so too does responsibility itself.^55^ But these nuances aside, both in the clinic and in the court, responsibility essentially tracks agency. Effective treatment presumes that patients are responsible and accountable for aspects of their pathology exactly in so far as they possess the cognitive and volitional capacities to which the justice model appeals to justify conviction and punishment. Thus far, the clinical model and the justice model accord. The difference between them rather lies in what each take attributions of responsibility to license with regard to our own attitudes, emotions and actions towards the patient or offender: whether or not, in virtue of being responsible and blameworthy for criminal or immoral conduct, we are licensed to affectively blame.

### B. Blame

What is it to blame another? Talk of blame is often ambiguous. When we say that another is ‘to blame’ we may mean one of three things:

- They are blameworthy.
- We should blame them.
- We actually do blame them.

These three propositions are distinct. (i) is a judgement about another. When a person is responsible for harm and has no excuse, they are blameworthy. But it is possible to make such a judgement about another, without also judging that we should blame them, let alone judging that we actually do. For instance, we might judge a historical figure from the distant past blameworthy for harm perpetrated, but we neither do blame them, nor judge that we should—the harm is too far removed.

(ii) is about us and what we should do. In this kind of context, ‘should’ can have three different meanings. First, we may be saying nothing more than that blame is *warranted* or *justified*: we should blame another, because they are blameworthy. If so, (ii) collapses into (i). Second, we may be saying that blame is *appropriate*, relative to various cultural norms governed by the nature of our role and relationship with the other, and the circumstances. For instance, it may be appropriate for victims to blame perpetrators for harm, when it is not appropriate for their legal advocates to do so. Third, we may be saying that blame is *desirable* relative to a given end: whether or not blame is warranted or appropriate to our role, relationship, and the circumstances, perhaps it would serve an instrumental purpose, such as deterrence. In all three senses, it may be true that we should blame another, and yet we find that we don’t. Perhaps we are simply too weary of battling or teaching this person, or fighting for social good: we are beyond caring at this stage to muster the energy to blame.

Finally, (iii) is about us and what we actually do. When others perpetrate wrongs or cause harm, we may find ourselves subject to a range of hostile, negative reactive attitudes and moral emotions directed towards them, such as hatred, anger, resentment, indignation, disgust, disapproval, contempt and scorn, and we may manifest these attitudes and emotions in any number of ways, including seeking retaliation, retribution, and vengeance, rejection and banishment from the community, and the withdrawal of basic respect and decency. Confronted by culpable wrongdoing or harm, we tend to feel that these hostile, negative attitudes and emotions are justified and appropriate because of what the person in question has done: we are entitled to feel and act in these ways, because they deserve it.^56^ Such attitudes and emotions may be rational in so far as they are based on accurate assessments of blameworthiness and are appropriate relative to our cultural norms. They may even on occasion serve a desired end.^57^ But, as with all reactive attitudes and moral emotions, they can also be irrational.

When others perpetrate harm, our shock and wound may make us quick to find someone to blame, so we have an object upon which we can vent our feelings and focus our sense of injustice and wrong. And we may do so even when some part of us knows that we shouldn’t: that the offending agent is not ultimately blameworthy, or that, even if they are, things are still not so black and white. These hostile, negative reactive attitudes and moral emotions can fly in the face of rational assessments of blameworthiness, cultural norms, and desirable ends. Our sense of our own entitlement to our wrath and righteous judgement of another’s conduct and character can itself be unjustly condemning and stigmatizing.^58^ And, even when it is just, there is yet the question of the potential harm it does to its object, and whether, in the grip of strong and potentially irrational attitudes and emotions, we are sufficiently able to calibrate them to what is justified or appropriate, never mind desirable: to be proportionate in our response to culpable wrongdoing or harm, and not to let it run away with us.

Call the simple judgement that another is blameworthy ‘detached blame’. Clinicians, like the courts, routinely form such judgements. Correspondingly, they may hold patients responsible for problematic behaviours, demand that they are accountable for them, and impose negative consequences. But the attitudes and emotions that accompany this practice are not hostile and negative. Rather, the clinical aim is to maintain throughout an attitude of concern, respect and compassion for the person, while nonetheless questioning, challenging and reproving their conduct: these attitudes underpin the clinical duty to help patients improve and recover. Call the range of hostile, negative attitudes and emotions that are typical responses to the perpetration of wrongdoing and harm, and to which we can feel, rationally or not, entitled, ‘affective blame’. The clinical aim is to avoid affective blame and its various manifestations. Where negative consequences are imposed, this is for the sake of psychiatric improvement, not out of retaliatory vengeance. Clinicians do not believe they are entitled to vent any hostile, negative attitudes and emotions on their patients. Concern and compassion, alongside basic respect and decency, is to be maintained, even in the face of blameworthiness.

With the distinction between detached and affective blame in hand, we can now see how responsibility and blame are conceptually distinct. Attributions of responsibility are essentially attributions of agency: the behaviour in question is simply a manifestation of the requisite cognitive and volitional capacities. When the behaviour is criminal or immoral and the agent has no justification or excuse, then they are blameworthy for it: there is criminal culpability or moral fault. But how we then react to such conduct is, to a significant degree, up to us. Accountability and negative consequences can be imposed *out of or in connection with affective blame*. Or they can be imposed with an attitude of concern, respect and compassion.

As we have seen, within the retributive tradition, punishment is typically conceived of as hard treatment, stigma, censure, exclusion, or the absence of mercy that is justified by blameworthy conduct. It is natural to understand these versions of ‘just deserts’ to invite if not indeed demand affective blame towards offenders. Recent research suggests that, as we would expect, prisoners, like patients, are highly sensitive to the affective tone of the environment in which they are punished.^59^ The clinical model offers us an alternative conception of punishment: consequences imposed in response to, by reason of, and in proportion to an agent’s blameworthy conduct, but not out of or in connection with affective blame. This demonstrates that there is no necessity in punishment being hard simply because it is justified by appeal to the offender’s moral agency; nor, *a fortiori*, even if it is negative, in it additionally being stigmatizing, exclusionary, merciless, or a form of lasting censure attaching to persons rather than conduct.

## 4. Taking the Clinical Model into the Legal Realm: Sentencing Procedures and the Courts

Affective blame is a typical human reaction to blameworthy conduct. Although the clinical stance of responsibility without blame is essential to effective treatment of disorders of agency and often achieved in practice, clinicians no doubt sometimes fail to keep affective blame at bay. But they are aided by various aims and procedures that guide clinical practice. In this section, we describe some of these aims and procedures and assess the appropriateness and feasibility of adapting them for use within the courts, particularly with respect to sentencing practices. Should we take the clinical model into this aspect of the legal realm, and if so, how do we do so?

One key feature of clinical practice that promotes detached as opposed to affective blame is the nature of the clinical role: the aim of clinical work is to help patients. This duty of care structures the relationship between clinician and patient, and, within many kinds of therapeutic groups, between patients themselves, and guides clinical engagement so that the therapeutic relationship is always at the fore. In so far as clinicians recognize the detrimental effect affective blame has on patients, they have reason to avoid it, or, if they fail entirely to avoid it, at least to avoid expressing it. Correspondingly, there are clear clinical guidelines and conventions that establish norms for how patients are spoken to and treated, which ensures a culture in which basic respect and decency is always expected, and often maintained. Lastly, it is no doubt possible to avoid affective blame precisely because of the acknowledgement and focus on detached blame and patient responsibility and accountability within various therapeutic processes: in the clinic as elsewhere, it is easier not to blame those who take responsibility for their problematic behaviour and ‘own up’ to their misconduct.

It is of course inconceivable that the criminal justice system could adopt the clinical aim to help and care in its pure form in relation to offenders: there should be no question about this impossibility. For criminal justice implies duties not only to offenders, but also to victims, the public and the law itself. These multiple duties are reflected in the multiple purposes of sentencing included in the UK Criminal Justice Act 2003 section 142: punishment, reduction of crime, reform and rehabilitation, public protection, and the making of reparation by the offender to those affected by the offence.

Nonetheless, these aims may be better served by attending to the model of responsibility without affective blame in the design and implementation of sentencing procedures. Reform, rehabilitation, reduction of crime, and, correspondingly, public protection that avoids such gross injustices as pre-emptive ‘treatment’ of the ‘dangerous’ and indefinite detention, may all be furthered by sentencing practices that follow the clinical model in holding offenders responsible and accountable without the stigmatizing and exclusionary effects of affective blame. For example, there is evidence that restorative justice, where offenders are held accountable for their behaviour and indeed required actively to take responsibility, with the aim not of condemnation, but of reintegration, can under certain conditions reduce crime and recidivism rates.^60^ While John Braithwaite’s early notion of ‘reintegrative shaming’ is open to question in terms of the potential association between ‘shaming’ and stigma or exclusion, the modulated statement of the aims of punishment in terms of reprobation twinned with—and tempered by—reintegration within his and Phillip Pettit’s more recent republican theory of punishment, bears an affinity with our argument here.^61^ In addition, when restorative justice includes dialogue between offender and victim, it may also contribute to the aim of reparation by the offender to those affected by the offence. The only current aim of sentencing that is not potentially served by taking the clinical model into the legal realm is punishment itself—if indeed the re-conception we have argued for is rejected and the demand for hardness and stigma maintained.

To insist on keeping affective blame at bay is not, therefore, to sacrifice the various instrumental goals of the penal system encoded in the purposes of sentencing: quite the reverse. As a result, just as clinicians are aided in avoiding affective blame by keeping the clinical aim to help at the forefront of their practice, so too the courts may be helped in avoiding affective blame by ensuring that they explicitly consider how the precise sentence and its execution can best serve the multiple purposes of sentencing already encoded in the law. This may help to redress a focus on punishment and proportionality, and create a better balance between the various ends sentencing is ideally supposed to achieve.

Similarly, as in the clinic, guidelines for how offenders are spoken to and treated in the courts, and vigilant monitoring of practices that may compromise basic respect and decency, are likely to be crucial. For example, consider victim impact statements. In their unconstrained form, as adopted in many jurisdictions in the United States, they deliberately invite, and provide a platform for, the expression of affective blame, which may bolster a punitive atmosphere in the court. In contrast, in England and Wales, victim impact statements that express sentencing preferences or blame are prohibited—constraints which implicitly recognize the importance of tempering the potential effect that affective blame can have on sentencing.^62^ As a society, we may want to affectively blame offenders. But prudentially, we may recognize that, given other ends we have, it is not desirable that we do so. And, given that serious and negative consequences would continue to be imposed in response to, by reason of, and in proportion to the offender’s blameworthy conduct according to the model we have presented, justice can be fully served in the absence of affective blame. Indeed, again as in the clinic, the imposition of these consequences may help us to avoid affective blame, in so far as they are designed to permit offenders to ‘own up’ to their misconduct.

A second key feature of clinical practice that promotes detached as opposed to affective blame is attention to patient history. As is well known, psychiatric disorders in general are associated with impoverished early childhood environments, and, of course, the ensuing psychosocial adversity, interpersonal and occupational problems, and stigma that is consequent upon poor mental health. In particular, personality and related disorders are associated with dysfunctional families, where there is breakdown, death, institutional care, and parental psychopathology; traumatic childhood experiences, with high levels of sexual abuse, and emotional and physical abuse or neglect; and social stressors, such as war, poverty and migration.^63^ Patients often come from harrowing backgrounds, impoverished of all goods, to an extent that can be unimaginable to people who have not experienced these kinds of conditions. As well as engaging patient agency, effective treatment can involve helping patients to explore their past and recognize its effects on their personality and their present experiences and behaviours, both as a way of coming to terms with the past, and as a way of developing skills needed to better manage the present. But, in attending to patients’ past history, clinicians and patients together gain understanding of why the patients are as they are. A fuller life story or narrative comes into view, in which the patient is seen not only as one who harms, but as one who has been harmed.

This capacity to see patients both as perpetrators and as victims can help clinicians avoid affective blame. It requires clinicians to keep in mind the whole of the person and the whole of their story, which undercuts a single attitude or emotion, forcing any affective blame to exist alongside compassion and understanding, and thereby at least reducing, if not outright extinguishing, its force. As Gary Watson has put this point in relation to the psychopath Robert Harris: ‘The sympathy towards the boy he was is at odds with outrage towards the man he is’.^64^ Indeed, there is evidence that this sort of contextualization may help to temper affective blame towards offenders. Research on social attitudes to offending consistently finds that more fully contextualized scenarios give rise to less punitive responses.^65^ In similar vein, studies of disadvantaged communities that are subject to high levels of both criminal victimization, and criminalization and punishment of their members, have demonstrated that they possess subtler attitudes to crime and punishment than those that typically characterize national criminal justice policies, formulated at a distance:^66^ local views typically combine a demand for penal accountability with a recognition that social interventions are also central to crime reduction.

Clearly, it is unlikely that the criminal justice system could ever concern itself with offenders’ past history and the context of the offence to the same degree as the clinical process: if nothing else, a life story or narrative takes a great deal of time to tell. Nonetheless, there is reason to think that such a concern should influence the attitude the courts take to offenders, to whatever extent is practically possible with the constraints of the courtroom. For, again, many offenders are also patients. Given the degree of psychiatric morbidity within the prison population, it is reasonable to conclude that many—particularly among the more serious offenders—are not only perpetrators, but also past victims of severe childhood psychosocial adversity, who not only have suffered terrible harm, but who also have not been given the opportunity to learn how to behave as moral citizens should.^67^

When children grow up in our midst subject to such conditions, arguably we as a society bear some responsibility for the harm inflicted on them if we fail to intervene. Our responsibility, in turn, may undercut our moral standing to affectively blame the adults these children become.^68^ There is therefore reason to hold that large-scale social institutions, like the criminal justice system, have a moral obligation to bear our collective failure to protect children and promote psychosocial equality in mind in the attitude taken to those who may have been victims before they became perpetrators. This is, to some degree, already recognized in sentencing practice: for example, pre-sentence reports addressing contextual factors such as these have long been a feature of the sentencing process in England and Wales.^69^ Furthermore, questions of responsibility aside, the moral standing to hold to account is also arguably premised on relatively equal relationships, and is hence undermined in radically unequal societies such as our own. Accordingly, this is yet another reason why we as a society may have a particular obligation to work for the reform and reintegration of offenders who are typically victims of social inequality and disadvantage prior to offending: it is not right to hold to account those who are not treated as equals without also working towards equal treatment for all.^70^ Hence not only does the criminal justice system have a host of instrumental reasons, given the purposes of sentencing, to avoid affective blame. It may also, as a large-scale social institution, have a moral obligation to do so.

Note, for clarity, that the appeal to adverse early environment and social inequality does not eliminate criminal responsibility or argue against accountability. Responsibility is attributed simply in virtue of agency. And accountability for blameworthy conduct, if we take the lesson to be learned from effective clinical treatment for disorders of agency seriously, not only serves justice, but also if imposed without affective blame equally serves the shared ends of psychiatric improvement and reform. Rather, the point is that a recognition of the harm done to many offenders as children, alongside the likelihood that they are victims more generally of social inequality and disadvantage, may appropriately undercut our sense that we are entitled to affectively blame, for as a society we should feel less entitled to condemn, stigmatize and marginalize those whom we have also wronged or deprived of social equality and opportunity, while maintaining our right to hold them responsible. Contextualizing sentencing decisions with respect to historical and psychosocial factors may, in short, help to keep affective blame at bay: ‘rotten social background’^71^ leaves basic criminal responsibility in place, but acknowledging its relevance may be the key to taking a more balanced view in sentencing and the execution of punishment, hence promoting prudential and moral ends alike.

## 5. Taking the Clinical Model into the Legal Realm: Prisons and the Aftermath of Punishment

In a broadly liberal, democratic society, respect and equality are ideally accorded to all. These moral and political values demand that punishment should proceed not only with humanity and dignity, but also with a view towards rehabilitation and reintegration of offenders into the moral community. To this end, we should try to create, as far as possible, non-stigmatizing and non-enduring practices of criminalization and punishment which do not, either materially or symbolically, permanently exclude offenders. This principle may seem particularly clear in relation to social attitudes to offenders after their sentence has been served: after all, they have paid their dues, as the saying goes, and accounted for their crime. But it bears also on the forms which punishments take, for there are powerful reasons to think that that form can make a long term difference to the prospects for future social inclusion and rehabilitation.

For instance, we might regard deliberately degrading aspects of prison regimes, such as identifying prisoners by number rather than name,^72^ or indeed the suspension of prisoners’ voting rights,^73^ which deprives prisoners of any voice in shaping the norms by which their community is governed^74^ as unjustifiably demeaning and exclusionary, with likely long-term effects on the future self-esteem and sense of civic membership of offenders. Similarly, branding, or indeed the scarlet letter which Hester Prynne was condemned to wear as perpetual punishment for her adultery in Nathaniel Hawthorne’s eponymous novel^75^—an eloquent exploration of the social and psychological upshot of stigmatization—are good examples of unjustifiable penalties, turning a form of corporeal punishment into lasting stigma. Long after the sentence has been served, the mark of past shame and ill character literally remains to be borne. Arguably, virtual forms of branding remain within contemporary society, despite the fact that corporeal branding is typically viewed with abhorrence. Under the UK Rehabilitation of Offenders Act 1974, although convictions punished by a non-custodial sentence, or a prison sentence of less than two and a half years, are ‘spent’ some years after that sentence is served, convictions punished by a prison sentence of more than two and a half years remain on the person’s record for the rest of their life. Furthermore, all offenders retain obligations to disclose even spent convictions, for all kinds of criminal offence, when applying for positions in a wide range of professions, including medicine, education, social work, accountancy and the law. Such practices undermine the possibility of genuine atonement, forgiveness, and full and lasting reintegration of offenders into the community: even when the punishment has been imposed and the sentence served, the crime is not forgotten.

Why do such stigmatizing and exclusionary practices persist? For it is evidently possible to design and implement forms of punishment which, like clinical practices, may also promote reform and rehabilitation: for example, community penalties or custodial sentences which involve meaningful work, restitution, education, training and therapeutic opportunities, and which are governed by respectful and humane attitudes and relations between staff and offenders.^76^ Many prisons in the Nordic countries enjoy regimes which offer such opportunities and possibilities even within this most serious of penal sanctions.^77^ Another model for such an approach is Therapeutic Community prisons, which have a strong history within the UK^78^ and a growing evidence base.^79^ Similarly, there is also a current initiative within the UK Ministry of Justice to develop a pilot programme of Psychologically Informed Planned Environments (PIPEs) that aims to implement some of the basic principles governing a more therapeutic approach within more mainstream custodial and probation settings.^80^ Unlike Therapeutic Communities, PIPEs do not provide therapeutic treatment, but they do employ therapeutic concepts and practices to structure the environment so as to promote reform and reintegration and guard against reinforcing criminal or immoral behaviour. Although outcomes are not yet known, and the social, political, and economic barriers to a more wide-scale implementation of Therapeutic Communities and PIPEs may be real, such initiatives, alongside Nordic prisons, nonetheless establish the clear possibility of creating penal practices radically different from those that currently pervade the criminal justice systems in the UK and the US. Hence a variety of possible models and knowledge-bases exist for the criminal justice system to draw upon should it choose to implement forms of punishment which avoid affective blame, and promote reform and rehabilitation instead.

One explanation for the persistent harsh and exclusionary penal practice in the US and UK may of course stem from public and judicial support for ‘three strikes’ sentencing laws and preventive measures based on public protection.^81^ But such support is likely to be driven at least in part by the tendency towards affective blame, together with the failure to distinguish clearly between it and a just demand for responsibility and accountability. Judgement of the conduct for which an offender is responsible is the business of the criminal process. A more generalized ‘judgmentalism’ or condemnation of the offender’s ‘bad character’ that is potentially lasting and stigmatizing, and hence undermining of the very possibility of any serious project of reform or reintegration, is not.^82^ The hostile, vengeful attitudes and emotions that are part and parcel of affective blame too easily allow us to focus less on the conduct, more on the person and their supposed nature. In their grip, we may feel entitled to write the offender off as ‘essentially’ of ‘bad character’ and to punish with cruelty and lasting ostracization and contempt.^83^ In other words, we may not only ‘hate the sin’ but also, in the grip of affective blame, come to hate the sinner. This can not only destroy the possibility of treating the offender with any concern, respect, or compassion, but also undermine any motivation we might otherwise harbour to work towards reform and reintegration. Quite generally, challenging the appropriateness of affective blame within law and society, and designing institutions and practices that aim to temper it, especially within custodial and probation contexts, may help to create non-stigmatizing and non-enduring forms of criminalization and punishment: to move us away from backward-looking retaliation and wrath, and towards a more humane and forward-looking attitude towards offenders.

## 6. Conclusion

We have argued that, despite appearances, the rehabilitative ideal and the justice model can be integrated. To do so, we must learn from the clinical model. Understanding that some crime is part of a psychopathology does not erode responsibility and accountability: the actions and omissions constitutive of disorders of agency are voluntary behaviour for which it is appropriate to hold offenders responsible and accountable. Indeed, within a clinical context, this is essential to effective treatment. There is thus scope for the criminal justice system to punish not only in the interest of justice, but equally in the interest of reform and rehabilitation: the justification of punishment need not limit its purposes to one or the other. But to achieve this, we must counter affective blame, and design institutions and practices that show concern, respect, and even on occasion compassion for the offender in imposing serious and negative consequences for criminal conduct, as opposed to institutions and practices that aim to serve hard treatment and stigma on those we believe to be deserving of condemnation and retaliation. In this way, justice for victims can be integrated with rehabilitation for offenders. So long as we continue to place the offender’s moral agency and corresponding responsibility for criminal conduct at the heart of penal philosophy, we can combine genuine respect and dignity for offenders with holding to account.

We have instrumental, moral, and political reasons for taking these aspects of the clinical model into the legal realm. Instrumentally, we have argued that the multiple purposes of sentencing are best served by procedures that aim to avoid affective blame. Morally, we have argued that the personal history and psychosocial disadvantage of many offenders may entail that, just as offenders have certain obligations to victims, we as a society have certain obligations to offenders. Finally, within a broadly liberal, democratic society, equal concern and respect are ideally accorded to all. These moral and political values demand that punishment should proceed not only with humanity and dignity, but with a view towards reform and reintegration of offenders into the moral community. Note that these values converge with the purposes of sentencing, to which we instrumentally appealed, already encoded in the law.

We thus have a host of good reasons to try so far as possible to design criminal justice institutions and practices that, even while upholding the intuition behind the justice model, whereby punishment is only justifiable in response to, by reason of, and in proportion to blameworthy criminal conduct, nonetheless punish without affective blame. Punishment may indeed consist of serious and negative consequences imposed in reaction to blameworthy conduct, but it need not—indeed should not—be ‘hard’ or exclusionary and stigmatizing. Drawing on clinical practice, we have suggested a variety of ways this re-conception of punishment might be put into practice. These include: fuller consideration of the various aims of sentencing, and relevant past history and psychosocial context and disadvantage of offenders within the courts; the opportunity for offenders to ‘take responsibility’ and acknowledge their offences and aim to make reparations to victims; the development of a culture of respect and humanity and the monitoring of language, attitudes, and practices that undermine such a culture, within courts, prisons, and agencies implementing community penalties; a commitment to forms of punishment which, like clinical practices, may also promote reform and rehabilitation, as exemplified in Nordic prisons, Therapeutic Community prisons in the UK, and more recently in PIPEs; and a recognition of the importance of the distinction between persons and their actions, to block a slide from appropriate judgement of misconduct to inappropriate judgements of ‘bad’ or ‘essential’ character that can carry enduring stigma and condemnation and destroy any motivation to work towards reform and reintegration of offenders. No doubt there are many other measures that might profitably be considered, some but not all of which may continue to draw on the clinical model. What we hope to have achieved here is a first step towards understanding why affective blame is not an inevitable component of the justice model, and the reasons we have, and steps we could take, to indulge in it both less frequently and less righteously.

## Footnotes

^1^ J Murphy, ‘Marxism and Retribution’ (1973) 2 P&PA 217; A von Hirsch, *Doing Justice* (Northeastern University Press 1976); A von Hirsch, *Censure and Sanctions* (Clarendon Press 1993); A von Hirsch and AJ Ashworth (eds), *Principled Sentencing* (2nd edn, Hart Publishing 1998); D McDermott, ‘The Permissibility of Punishment’ (2001) 20 Law and Philosophy 403.

^2^ H Pickard, ‘Mental Illness is indeed a Myth’ in MR Broome and L Bortolotti (eds), *Psychiatry as Cognitive Neuroscience *(OUP 2009) 83; S Pearce and H Pickard, ‘Finding the Will to Recover: Philosophical Perspectives on Agency and the Sick Role’ (2010) 36 (12) J Medical Ethics <http://jme.bmj.com/content/36/12/831> accessed 31 July 2010; H Pickard, ‘What is Personality Disorder?’ (2011) 18 Philosophy, Psychiatry, Psychology 181.

^3^ H Pickard, ‘Responsibility Without Blame: Philosophical Reflections on Clinical Practice’ in TWM Fulford and others (eds), *The Oxford Handbook of Philosophy of Psychiatry* (OUP forthcoming).

^4^ ibid.

^5^ ibid.

^6^ S Pearce and H Pickard, ‘The Moral Content of Psychiatric Treatment’ (2009) 195 British J Psychiatry 281 and Pearce and Pickard (n 2); Pickard, ‘Responsibility Without Blame: Empathy and the Effective Treatment of Personality Disorder’ (n 3).

^7^ Indeed one of us has defended a partially consequentialist theory of punishment: N Lacey, *State Punishment: Political Principles and Community Values *(Routledge 1988).

^8^ J Rawls, *A Theory of Justice* (Harvard University Press 1971).

^9^ B Wootton, *Social Science and Social Pathology* (George Allen and Unwin 1959); B Wootton, *Crime and the Criminal Law* (Stevens and Sons 1963).

^10^ HLA Hart, in J Gardner (ed), *Punishment and Responsibility* (first published 1968, 2nd edn, OUP 2008).

^11^ For an example from the United States, see American Friends Service Committee, *Struggle for Justice* (Hill and Wang 1971); F Allen, *The Decline of the Rehabilitative Ideal *(Yale University Press 1981); from the UK, cf Wootton (n 9).

^12^ M Tonry (ed), *Retributivism has a Past: Has it a Future?* (OUP 2011).

^13^ American Friends Service Committee (n 11); von Hirsch, *Doing Justice* (n 1).

^14^ Hart (n 10); Lacey (n 7); von Hirsch, *Censure and Sanctions* (n 1); J Braithwaite and P Pettit, *Not Just Deserts* (OUP 1990); RA Duff, *Punishment, Communication and Community* (OUP 2001). Note however that these more complex views take different positions on the relationship between criminal responsibility and blameworthiness. For example, while Hart is explicitly concerned to distinguish legal from moral responsibility (Hart (n 10) 210ff), Duff emphasizes the resonance between the two in his vision of criminal trial and punishment as means of ‘answering for crime’ understood as wrongdoing (RA Duff, *Answering for Crime: Responsibility and Liability in the Criminal Law* (Hart Publishing 2007)).

^15^ B Hudson, *Justice Through Punishment* (Macmillan 1987).

^16^ R Martinson, ‘What Works?—Questions and Answers about Prison Reform’ (1974) 35 Public Interest 22; L Wilkins, *Evaluation of Penal Measures* (Random House 1969).

^17^ American Friends Service Committee (n 11); von Hirsch, *Doing Justice* (n 1).

^18^ D Garland, *The Culture of Control* (OUP 2001); D Garland (ed), *Mass Imprisonment in the United States: Social Causes and Consequences* (Sage 2001); J Whitman, *Harsh Justice* (OUP 2003); J Pratt, *Penal Populism* (Routledge 2006); R Reiner, *Law and Order* (Polity Press 2007); J Simon, *Governing Through Crime: How the War on Crime Transformed American Democracy and Created a Culture of Fear* (OUP 2007); N Lacey, *The Prisoner’s Dilemma: Political Economy and Punishment in Contemporary Democracies* (CUP 2008); AJ Ashworth and L Zedner, ‘Defending the Criminal Law’ (2008) 2 Crim L and Philosophy 21 and Ashworth and Zedner, ‘Preventive Orders: A Case of Undercriminalization’ in RA Duff and others (eds), *The Boundaries of the Criminal Law* (OUP 2010); L Zedner, ‘Security, the State and the Citizen: the Changing Architecture of Crime Control’ (2010) 13 New Crim L Rev 379.

^19^ N Lacey, ‘Political Systems and Criminal Justice: The Prisoner’s Dilemma after the Coalition’ (2012) CLP <http://clp.oxfordjournals.org/content/early/2012/03/07/clp.cus002.full.pdf> accessed 8 March 2012.

^20^ AJ Ashworth, *Sentencing and Criminal Justice* (5th edn, CUP 2010); AJ Ashworth and A von Hirsch, *Proportionate Sentencing: Exploring the Principles* (OUP 2005).

^21^ A von Hirsch and N Jareborg, ‘Sweden’s Sentencing Statute Enacted’ (1989) Crim LR 275; J Pratt ‘Scandinavian Exceptionalism in an Era of Penal Excess’, Part I: ‘The Nature and Roots of Scandinavian Exceptionalism’ (2008) 47 British J Crim 119; ‘Scandinavian Exceptionalism in an Era of Penal Excess’, Part II: ‘Does Scandinavian Exceptionalism Have a Future?’ (2008) 48 British J Crim 275.

^22^ M Matravers, ‘Is Twenty-first Century Punishment Post-desert?’ in Tonry (ed) (n 12) 30; see also PH Robinson, ‘Competing Conceptions of Modern Desert: Vengeful, Deontological, and Empirical’ (2008) 67 CLJ 67, 145.

^23^ RA Duff, *Punishment, Communication and Community* (n 14).

^24^ M Matravers, ‘Duff on Hard Treatment’ in R Cruft, MH Kramer and MR Reiff (eds), *Crime, Punishment and Responsibility: The Jurisprudence of Antony Duff* (OUP 2011).

^25^ RA Duff (n 14) 77–79. Arguably this is an upshot of his resistance to giving prudential or consequentialist considerations a place in his account.

^26^ J Tasioulas, ‘Where is the Love? The Topography of Mercy’ in Croft, Kramer and Reiff (eds) (n 24).

^27^ von Hirsch, *Censure and Sanctions* (n 1).

^28^ D Husak, ‘Lifting the Cloak: Preventive Detention as Punishment’ (2011) 48 San Diego L Rev 1173, 1182.

^29^ D McDermott, ‘The Permissibility of Punishment’ (n 1) 428–29.

^30^ Lacey (n 18).

^31^ National Offender Management Strategy (NOMS), ‘Working with Personality Disordered Offenders: A Practitioner’s Guide’ (NOMS 2011).

^32^ National Institute of Mental Health in England (NIMH(E)), *Personality Disorder: No Longer a Diagnosis of Exclusion *(NIMH(E) 2002).

^33^ H Pickard, ‘What Aristotle can Teach us about Personality Disorder’ (2011) commissioned by the National Personality Disorder Website <http://www.personalitydisorder.org.uk/news/wp-content/uploads/article.pdf>; H Pickard, ‘The Purpose in Chronic Addiction’ (2012) 3 Am J Bioethics Neuroscience 40; H Pickard and S Pearce, ‘Addiction in Context: Philosophical Lessons from a Personality Disorder Clinic’ in N Levy (ed), *Addiction and Self-Control* (OUP forthcoming).

^34^ These include, for instance, many forms of (1) cognitive-behavioural therapy: M Lineham and L Dimeff, ‘Dialectical Behavioural Therapy in a Nutshell’ (2001) 34 The California Psychologist 10; K Davidson, *Cognitive Therapy for Personality Disorders* (2nd edn, Routledge 2008); N Blum and others, ‘Systems Training for Emotional Predictability and Problem Solving (STEPPS) for Outpatients With Borderline Personality Disorder: A Randomized Controlled Trial and 1-Year Follow-Up’ (2008) 165 Am J Psychiatry 468; (2) motivational interviewing: A Bandura, *Self Efficacy: The Exercise of Control* (Worth Publishers 1997); S Rollnick and WR Millner, ‘What is Motivational Interviewing?’ (1995) 23 Behavioural and Cognitive Psychotherapy 325; (3) stop-and-think training: M McMurran and others, ‘Stop and Think!: Social Problem Solving Therapy with Personality Disordered Offenders’ (2001) 11 Criminal Behaviour and Mental Health 273; (4) all varieties of exposure therapy: P de Silva, ‘Obssessive Compulsive Disorder’ in A Carr and M McNulty (eds), *The Handbook of Adult Clinical Psychology* (Routledge 2006); (5) emotional intelligence: D Goleman, *Working with Emotional Intelligence* (Bantam Books 1998); (6) mentalization-based therapy: JG Allen and P Fonagy, *Handbook of Mentalization-based Treatment* (Wiley 2006); AW Bateman and P Fonagy, *Mentalization-based Treatment for Borderline Personality Disorder: A Practical Guide* (OUP 2006); P Fonagy and A Bateman 2006, ‘Progress in the Treatment of Borderline Personality Disorder’ (2006) 188 British J Psychiatry 1; and (7) Therapeutic Communities (often considered the treatment of choice for personality disorders): J Lees, N Manning and B Rawlings, *Therapeutic Community Effectiveness: A Systematic International Review of Therapeutic Community Treatment for People with Personality Disorders and Mentally Disordered Offenders* (York Publishing Services Ltd 1999). Note that these have a history of use within prisons: M Parker, *Dynamic Security: The Democratic Therapeutic Community in Prisons* (Jessica Kingsley Publishers 2007).

^35^ Pickard (n 3).

^36^ E Genders and E Player, *Grendon: A Study of a Therapeutic Prison* (OUP 1995); J Shine (ed), *A Compilation of Grendon Research* (HM Prison Grendon 2000).

^37^ For further discussion see Pickard (n 3); Pickard, ‘The Purpose in Chronic Addiction’ (n 33); Pickard and Pearce, ‘Addiction in Context: Philosophical Lessons from a Personality Disorder Clinic’ (n 33); S Pearce and H Pickard, ‘How Therapeutic Communities Work: Specific Factors related to Positive Outcome’ (2012) Intl J Social Psychiatry (published online 20 July 2012 DOI: 10.1177/0020764012450992).

^38^ P Gilbert, *Compassion Focused Therapy *(Routledge 2010). See too K Neff, *Self-Compassion* (Hodder and Stoughton 2011).

^39^ One obvious kind of question, for instance, is the nature of the mechanisms: to what degree are the underlying psychological processes conscious or unconscious, innate or learned, rational or affective?

^40^ PF Strawson, ‘Freedom and Resentment’ (1962) 48 Proceedings of the British Academy 1.

^41^ G Watson, ‘Responsibility and the Limits of Evil’ in his *Agency and Answerability: Selected Essays* (OUP 2004) 220.

^42^ See eg M Zimmerman, *An Essay on Moral Responsibility *(Roman and Littlefield 1988); RJ Wallace, *Responsibility and the Moral Sentiments* (Harvard University Press 1994); JM Fischer and M Ravizza, *Responsibility and Control: A Theory of Moral Responsibility* (CUP 1998).

^43^ D Pereboom, *Living Without Free Will* (CUP 2001) 87.

^44^ cf A Smith, ‘On Being Responsible and Holding Responsible’ (2007) 11 J Ethics 465.

^45^ For further discussion of the difficulties faced by this idea of responsibility in accounting for clinical practice, see Pickard (n 3).

^46^ cf Hart (n 10).

^47^ Aristotle, ‘Eudemian Ethics’ in J Barnes (ed), *The Complete Works of Aristotle* (J Solomon tr, OUP 1984).

^48^ T Hobbes, ‘Treatise: Of Liberty and Necessity’ in V Chappell (ed), *Hobbes and Bramhall on Liberty and Necessity* (CUP 1999).

^49^ D Hume, in LA Selby Bigge (ed), *Enquiry Concerning Human Understanding* (OUP 1975).

^50^ T Reid, in W Hamilton (ed), *The Works of Thomas Reid Vol. 2 *(Thoemmes Press 1994).

^51^ I Kant, in T Greene and H Hudson (trs), *Religion Without the Bounds of Reason Alone* (Harper and Row 1960).

^52^ For a historical survey and contemporary defence of this view see H Steward, ‘Fairness, Agency, and the Flicker of Freedom’ (2009) 43 Nous 64; cf M Alvarez, ‘Actions, thought-experiments and the “Principle of Alternate Possibilities”’ (2009) 87 Australasian J Philosophy 61. For a more detailed historical discussion of the idea of free will in the ancient world see S Bobzien, ‘The Inadvertent Conception and Late Birth of the Free-Will Problem’ (1998) 43 Phronesis 133.

^53^ cf Hart (n 10); R Holton, ‘Disentangling the Will’ in R Baumeister, A Mele and K Vohs (eds), *Free Will and Consciousness: How Might They Work?* (OUP 2010). For an important analysis of the nature of such capacities, see M Smith, ‘Rational Capacities, or: How to Distinguish Recklessness, Weakness, and Compulsion’ in S Stroud and C Tappolet (eds), *Weakness of Will and Varieties of Practical Irrationality* (OUP 2003).

^54^ See eg J Gardner, *Offences and Defences* (OUP 2007); J Horder, *Excusing Crime* (OUP 2004).

^55^ For discussion on this question in relation to the law’s treatment of personality disordered offenders, see J Peay, ‘Personality Disorder and the Law: Some Awkward Questions’ (2011) 18 Philosophy, Psychiatry, Psychology 231 and W Sinnott-Armstrong, ‘Personality Disorder and Responsibility: Learning from Peay’ (2011) 18 Philosophy, Psychiatry, Psychology 245.

^56^ Pickard (n 3).

^57^ V McGeer, ‘Civilising Blame’ in DJ Coates and NA Tognazzini (eds), *Blame: Its Nature and Norms *(OUP 2013)*.*

^58^ N Lacey, ‘The Resurgence of Character: Criminal Responsibility in the Context of Criminalisation’ in RA Duff and S Green (eds), *Philosophical Foundations of Criminal Law* (OUP 2011).

^59^ See eg A Liebling, ‘Moral Performance, Inhuman Treatment and Degrading Treatment and Prison Pain’ (2011) 13 Punishment and Society 530.

^60^ J Braithwaite, *Crime, Shame, and Reintegration* (CUP 1989) and J Braithwaite, *Restorative Justice and Responsive Regulation* (OUP 2002); L Sherman and H Strang, *Restorative Justice: The Evidence* (The Smith Institute 2007); G Robinson and J Shapland, ‘Reducing Recidivism: A Task for Restorative Justice?’ (2008) 48 British J Crim 337; J Shapland, G Robinson and A Sorsby, *Restorative Justice in Practice: Evaluating What Works for Victims and Offenders* (Routledge 2011).

^61^ Braithwaite and Pettit (n 14) 88–92. For a persuasive appraisal of the literature on reintegrative shaming, including a discussion of the psychological evidence on potential dangers implicit in the institutionalization of shaming, see N Harris and S Maruna, ‘Shame, Shaming and Restorative Justice’ in D Sullivan and L Tifft (eds), *Handbook of Restorative Justice: A Global Perspective* (Routledge 2006) 452–62. Harris and Maruna argue for an understanding of restorative justice as ‘management’ of the shame which inevitably arises as a by-product of calling an offender to account, within a practice which disciplines the negative, exclusionary potential of shame in favour of its remorse-promoting aspects (see especially 454–6, 459–60). We are sympathetic to the view that certain kinds of restorative justice may have some capacity to ‘manage’ and marshal complex negative emotions: but we are strongly of the view that criminal courts are neither suitable nor legitimate places for this task.

^62^ MD Dubber, *Victims in the War on Crime: The Use and Abuse of Victim’s Rights *(NYU Press 2006).

^63^ J Paris, ‘Psychological Adversity’ in WJ Livesley (ed), *Handbook of Personality Disorders* (Guildford Press 2001).

^64^ Watson (n 41) 244.

^65^ J Roberts and M Hough (eds), *Changing Attitudes to Punishment: Public Opinion, Crime and Justice* (Willan Publishing 2002).

^66^ L Miller, *The Perils of Federalism: Race, Poverty, and the Politics of Crime Control* (OUP 2008).

^67^ Pickard, ‘Mental Illness is indeed a Myth’ (n 2) and ‘What Aristotle Can Teach us About Personality Disorder’ (n 33); Prison Reform Trust, *Justice for Women* (Prison Reform Trust 2000).

^68^ cf T Scanlon, *Moral Dimensions* (Harvard University Press 2008).

^69^ Ashworth (n 20) 378–80.

^70^ cf Duff, *Punishment, Communication and Community *(n 14) ch 5; Braithwaite and Pettit (n 14) 182 ff. We here use ‘equal treatment’ synonymously with ‘treatment as an equal’, implying a precept of equal concern and respect as defended in the early work of RM Dworkin (see eg *Taking Rights Seriously* (Duckworth 1977). We do not, therefore, mean to imply that ‘treatment as an equal’ necessarily equates to equal treatment in the sense of an equal distribution of welfare, outcomes or resources; but, while we cannot argue in full for this position here, we do hold that certain degrees of unjust distributive inequality may be inconsistent with delivery of equal treatment in our sense.

^71^ R Delgado, ‘“Rotten Social Background”: Should the Criminal Law Recognize a Defense of Severe Environmental Deprivation?’ (1985) 3 L and Inequality 9, 54–55; PH Robinson, ‘Are We Responsible for Who We Are? The Challenge for Criminal Law Theory in the Defences of Coercive Indoctrination and “Rotten Social Background”’ (2011) 2 Alabama Civil Rights and Civil Liberties L Rev 53.

^72^ Whitman (n 18); conversely, French and German protocols designed to ensure respectful treatment of prisoners, such as addressing them formally by their title and surname, are good examples of practices consistent with the ideal of responsibility without blame.

^73^ In *Hirst v the United Kingdom (No 2)* (2006) 42 EHRR 41 the European Court of Human Rights held that the UK’s blanket exclusion of convicted prisoners from the franchise was incompatible with the European Convention on Human Rights; in an Interim Resolution of 2009, recalling that the UK’s ‘general, automatic and indiscriminate restriction on the right of convicted prisoners in custody to vote, fell outside any acceptable margin of appreciation and was incompatible with Article 3 of Protocol No. 1 to the Convention’ the court deplored the UK government’s delay in implementing the 2005 judgement. (Council of Europe Committee of Ministers, Interim Resolution CM/ResDH (2009) 160). For a different view, see P Ramsay, ‘Voters should not be in Prison’ Critical Review of Internationalfund Social and Political Philosophy (Special Issue, 2013).

^74^ L Lazarus, *Contrasting Prisoners' Rights* (OUP 2004); Pratt, ‘Scandinavian Exceptionalism’ Parts I and II (n 21).

^75^ N Hawthorne, *The Scarlet Letter* (first published 1850, Oxford World’s Classics, OUP 2007).

^76^ Sadly, current retrenchments in public spending in the UK appear to be putting just such practices under threat: see M O’Hara, ‘A Costly Decision for Prisoner Rehabilitation Support? *The Guardian* (London, 26 July 2011) <http://www.guardian.co.uk/society/2011/jul/26/latchmere-house-prisoner-rehabilitation-support> accessed 26 July 2011.

^77^ See J Pratt and A Eriksson, *Contrasts in Punishment: An Explanation of Anglophone Excess and Nordic Exceptionalism* (Routledge 2013); Pratt, ‘Scandinavian Exceptionalism’ Parts I and II (n 21).

^78^ See eg Genders and Player (n 36) and Parker (n 38). A somewhat different example, but one that also deployed a model compatible with responsibility without blame, was the Special Unit established at Barlinnie Prison in 1973, as recounted in Jimmy Boyle’s *A Sense of Freedom* (Pan 1977). It is relevant to the historical story reviewed in the early part of this paper that the Unit was closed in 1994.

^79^ See eg Shine (n 36).

^80^ NOMS, PIPEs: <http://www.personalitydisorder.org.uk/news/wp-content/uploads/DHNOMS_PIPE2011_Info.pdf>; N Benefield and R Haigh ‘Providing Psychologically Informed Environments in Residential Settings’ (MS).

^81^ Ashworth and Zedner, ‘Defending the Criminal Law’ and ‘Preventive Orders’ (n 18).

^82^ Lacey (n 58); G Watson, ‘Standing in Judgment’ in DJ Coates and NA Tognazzini (eds), *Blame: Its Nature and Norms *(OUP 2013).

^83^ Lacey (n 58).

